# Painful Ophthalmoplegia: Aspergillosis, Tolosa-hunt and other Causes

**DOI:** 10.5334/jbsr.1496

**Published:** 2018-02-27

**Authors:** Antoine Liesse, David Salerno

**Affiliations:** 1 CHU Liège, BE; 2 CSL Arlon, BE

**Keywords:** Painful ophthalmoplegia, Tolosa Hunt, aspergillosis, orbital apex, cavernous sinus

Dear Editor,

An 81-year-old man was referred to our department for investigation of right-sided painful ophthalmoplegia. He had a history of chronic obstructive pulmonary disease and benign prostatic adenoma. The anamnesis revealed the use of mycophenolate mofetil (an immunosuppressant drug) for a few months, following a suspicion of rheumatoid mono-arthritis. Clinical evaluation showed a reddish right upper eyelid, chemosis of the right eye, a slight proptosis and diplopia caused by a palsy of the right abducens nerve (VI). The pupils were equal and reactive. Visual acuity was normal.

Orbital and skull-base MRI (Figure 1) was performed, showing a soft-tissue process infiltrating the fat in the orbital apex, extending through the optic canal and the superior orbital fissure towards the right cavernous sinus. The lesion was hyperintense on T2-weighted imaging (WI) and isointense to the orbital muscles on T1-WI before contrast administration and avidly enhancing after intravenous gadolinium administration on T1-WI images with fat suppression. The eyeballs, the optic nerves and the extraocular muscles were normal. The skull-base, the cisternal segments of the cranial nerves, the sella turcica and the optic chiasm were unremarkable. There was neither signal abnormality of the cerebral parenchyma, nor parenchymal or meningeal enhancing lesions following gadolinium administration.

**Figure 1:**
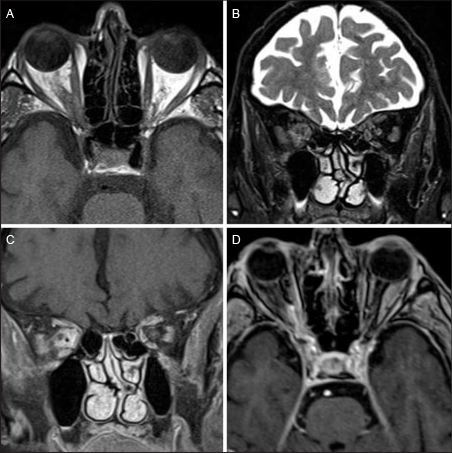
**A** – Axial T1 TSE image. **B** – Coronal T2 weighted-image with fat-suppression. **C** – Coronal T1 Dixon gadolinium-enhanced image. **D** – Axial gadolinium-enhanced T1 image with fat-suppression.

The orbital apex is a region loaded with muscular, nervous and vascular structures, amongst others, including the origin of the extraocular muscles as well as the optic nerve. The cavernous sinus is the structure of the skull-base with the densest concentration of critical vascular and neural structures. It contains the oculomotor, trochlear and abducens nerves, as well as the first and second divisions of the trigeminal nerve, the internal carotid artery and its nervous plexus. These two anatomical regions therefore establish important links between the skull-base and the orbital and facial areas, leading to various clinical symptoms depending on the involvement of each structure in the pathological process [1].

A radioclinical presentation such as the one described in our patient raises the possibility of multiple etiologies: vascular (superior ophthalmic vein thrombosis, carotido-cavernous fistula, arteritis), inflammation (granulomatous disease including sarcoidosis, inflammatory pseudotumor, or idiopathic in Tolosa-Hunt), infection (aspergillosis) or tumour (primary or metastatic) [2].

Tolosa-Hunt syndrome is an idiopathic granulomatous inflammation of the cavernous sinus and/or the orbital apex/superior orbital fissure [3]. The diagnostic criteria consist of (1) steady, gnawing pain behind the eye with ophthalmoplegia, (2) involvement of nerves running through the cavernous sinus, (3) duration of symptoms of days to weeks, (4) spontaneous remissions, sometimes with residual deficit, (5) recurrent attacks at intervals of months or years and (6) exclusion of all other disease process [4].

Considering the use of immunosuppressant drugs by our patient and the absence of other significant systemic signs, the presumptive diagnosis of orbital aspergillosis was retained. It was therefore decided to start a treatment with oral administration of voriconazole (an azole antifungal agent). Within a few days, ocular symptoms and pain decreased.

Following the increasing use of antibiotics, corticosteroids, chemotherapy, and due to the increasing prevalence of diabetes in the population, more and more cases of orbital aspergillosis have been reported. It is thereby a diagnosis that we should not neglect when confronted to a painful ophthalmoplegia.

It is nonetheless important not to disregard other serious and possibly life-threatening conditions such as rapidly evoluting vascular abnormalities (aneurysms and fistulae) or tumours (orbital lymphomas, metastasis) [5].

## References

[1] Shama, S and Gheida, U. Superior orbital fissure and its mimics: What the radiologist should know? Egyptian Journal of Radiology and Nuclear Medicine. 2012; 43: 589–594. DOI: 10.1016/j.ejrnm.2012.09.009

[2] Marcet, M, Yang, W, Albert, D, et al. Aspergillus infection of the orbital apex masquerading as Tolosa-Hunt syndrome. Arch Ophthalmol. 2007; 125(4): 563–566. DOI: 10.1001/archopht.125.4.563 17420381

[3] Tolosa, EJ. Periarteritic lesions of the carotid siphon with clinical features of carotid intraclinoid aneurysmus. J Neurol Neurosurgery Psychiatry. 1954; 17: 300–302. DOI: 10.1136/jnnp.17.4.300 PMC503202 13212421

[4] Hunt, WE and Lefever, H. Painful ophthalmoplegia: Its relation to indolent inflammation of the cavernous sinus. Neurology. 1961; 11: 56–62. DOI: 10.1212/WNL.11.1.56 13716871

[5] Khera, PS, Singh, S, Chowdhury, V and Dixit, R. Tolosa Hunt Syndrome: A case report. Ind J Radiol Imag. 2006; 16(2): 175–177. DOI: 10.4103/0971-3026.29081

